# Haemoptysis in Askin's Tumour: Intrapulmonary Presentation of an Extrapulmonary Tumour

**DOI:** 10.7759/cureus.25267

**Published:** 2022-05-24

**Authors:** Ujjawal Khurana, Roshny J, Hemlata Panwar, Sramana Mukhopadhyay, Alkesh Khurana

**Affiliations:** 1 Pathology and Lab Medicine, All India Institute of Medical Sciences, Bhopal, Bhopal, IND; 2 Pathology and Lab Medicine, AIl India Institute of Medical Sciences, Bhopal, Bhopal, IND; 3 Pulmonary and Critical Care Medicine, All India Institute of Medical Sciences, Bhopal, Bhopal, IND

**Keywords:** lung tumor, haemoptysis, ewing sarcoma family of tumors(esft), cd99, askin tumor

## Abstract

Ewing’s sarcoma (ES) is a small round cell sarcoma arising in the bone or soft tissue. Ewing’s sarcoma/primitive neuroectodermal tumours (PNET) of the thoracopulmonary region is called Askin’s tumour. The common clinical presentations described for this extrapulmonary tumour are fever, chest wall mass with or without pain, dyspnea, and cough. Very few cases of Askin’s tumour have been reported with haemoptysis as the initial presentation, which is usually a presentation of intrapulmonary lesions. A 22-year-old male presented to the emergency department with complaints of haemoptysis, mild chest pain, and swelling on the right side of the chest wall. Radiological investigations showed a soft tissue mass measuring 13 cm × 11 cm × 10 cm in the right thoracic region, causing the destruction of the second rib. Histopathological examination showed the presence of a malignant small round cell tumour. Immunohistochemistry (IHC) analysis showed the tumour to be positive for CD99, NKX 2.2, and MIC2. The final diagnosis of Askin’s tumour of the thoracopulmonary region was given. The case is being reported in view of the rare type of clinical presentation.

## Introduction

Ewing’s sarcoma (ES) is a malignant small round cell tumour with characteristic molecular findings and varying neuroectodermal differentiation. It is mostly skeletal but can be extraskeletal, with the incidence of the latter reported to be around 12-13% [1,2]. Extraskeletal Ewing's sarcoma/primitive neuroectodermal tumours (PNET) are rare, aggressive variants of small round cell tumours with a postulated primary origin from neural crest cells. The common locations of the extraosseous type include the paravertebral spaces, lower extremities, and rarely the skin and chest wall. According to the World Health Organization, classic Ewing's sarcoma, extraskeletal Ewing's sarcoma, and PNETs constitute the Ewing's sarcoma family of tumours (ESFT). 

Ewing’s sarcoma/PNET tumour of the thoracopulmonary region is conventionally called Askin’s tumour [1,3]. In 1979, Askin et al. compiled a list of 20 cases of malignant small round cell tumours of the thoracopulmonary region in childhood, which later came to be known as "Askin’s tumour."

The common clinical presentations of these tumours are fever, chest wall mass with or without pain, dyspnea, cough, etc. Very few cases of Askin’s tumour have been reported with haemoptysis as the initial presentation [4]. Herein, we present a case of Askin’s tumour in a 22-year-old male who presented with haemoptysis, which is the usual presentation of intrapulmonary lesions and tumours.

## Case presentation

A male in his early second decade came to the emergency department with a history of swelling in the right side of the chest wall for 20 days and with a presenting complaint of haemoptysis and shortness of breath for 4 days. There was no associated loss of weight or loss of appetite. He had no comorbidities. He was an occasional smoker. On examination, a single, firm swelling was noted on the right side of the chest wall at the level of the second and third ribs.

**Figure 1:**
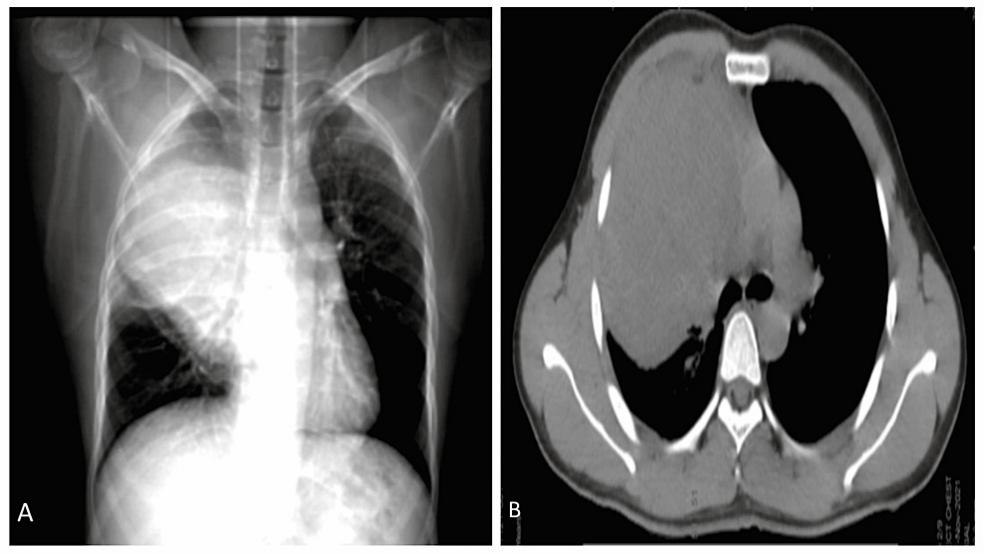
Radiological images (A) Chest X-ray shows large, homogenous soft tissue density mass lesion occupying the right upper lobe region. (B) CECT scan shows a large mass in the right lung region which reveals rib destruction and extends across the chest wall. The mass shows peripheral necrotic areas.

Radiological investigations of the swelling in the chest wall were done. A soft tissue mass in the right infraclavicular region involving the deep muscular plane and the intrathoracic region was noted on the chest X-ray (Figure 1A). CECT chest (Figure 1B) revealed a large heterogeneously enhancing soft tissue mass measuring 13 cm × 11 cm × 10 cm in the right thoracic region, causing destruction of the second rib anteriorly and extending into the extra-thoracic space in the anterior chest wall. The mass caused compression and displacement of the mediastinum. The PET CT chest showed the soft tissue mass to be involved in the second rib anteriorly, causing erosion of the third rib. No other skeletal abnormalities and no definite metastatic deposits were identified. With a left ventricular ejection fraction (LVEF) of 55-60%, the echocardiogram showed that the heart's systolic function was normal.

A complete haemogram revealed haemoglobin (Hb) of 15.5 g/dl, TLC of 8500 cells/cumm, and a platelet count of 3.25 lakhs/cumm. Coagulation studies showed a mildly increased prothrombin time of 18 seconds (normal: 12-16 seconds), a reduced prothrombin index of 72.22, a prothrombin ratio of 1.38, and an INR of 1.43. Biochemical tests showed normal serum creatinine of 0.80 mg/dl, normal total bilirubin of 0.69 mg/dl, normal direct bilirubin of 0.17 mg/dl, normal indirect bilirubin of 0.51 mg/dl, normal SGOT of 29 IU/L, normal SGPT of 23 IU/L, normal alkaline phosphatase of 122 IU/L, normal serum sodium of 137 mmol/L, S. potassium of 4.40 mmol/L and S. chloride of 102 mmol/L.

**Figure 2:**
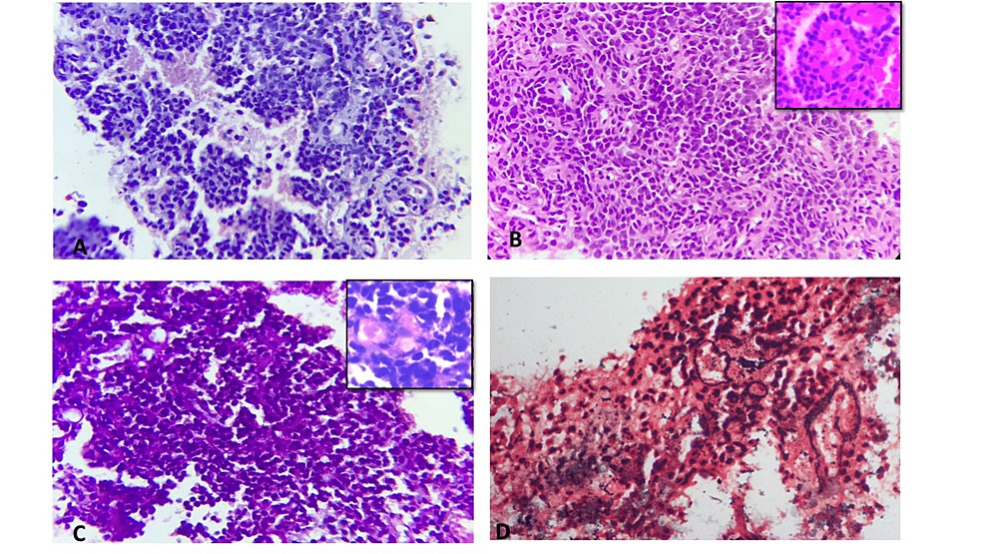
Histopathology images (A) Cellular and richly vascular tumour arranged in sheets and peritheliomatous pattern showing interspersed necrotic areas. (H & E; ×400). (B) Tumour cells are comprised of a uniform population of monomorphic small round cells with hyperchromatic nuclei, scant eosinophilic cytoplasm (H & E; ×400), and at places surrounding avascular matrix-forming pseudorosettes. (Inset C) Tumour cells show cytoplasmic magenta colour on PAS stain (PAS; ×400) which was diastase sensitive as shown in inset (PAS-D; ×400). (D) Reticulin stain highlights the rich vasculature of the tumour (reticulin; ×400).

A USG-guided biopsy from the soft tissue lesion was sent for histopathological examination. The tissue biopsy revealed a richly vascular tumour showing a tumour arranged in sheets, a peritheliomatous pattern with interspersed necrotic areas (Figure 2A). The tumour cells were small, round with hyperchromatic nuclei, inconspicuous nucleoli, and a scant to moderate amount of pale eosinophilic cytoplasm. Tumour cells surrounding an avascular matrix and forming pseudorosettes were seen (Figure 2B). Mitosis, apoptosis, and necrosis were noted. Tumour cells were periodic acid-Schiff (PAS)-positive and diastase sensitive, indicating the presence of glycogen granules (Figure 2C). Reticulin stain highlighted the vascularity and loss of reticulin meshwork in the necrotic areas (Figure 2D).

**Figure 3:**
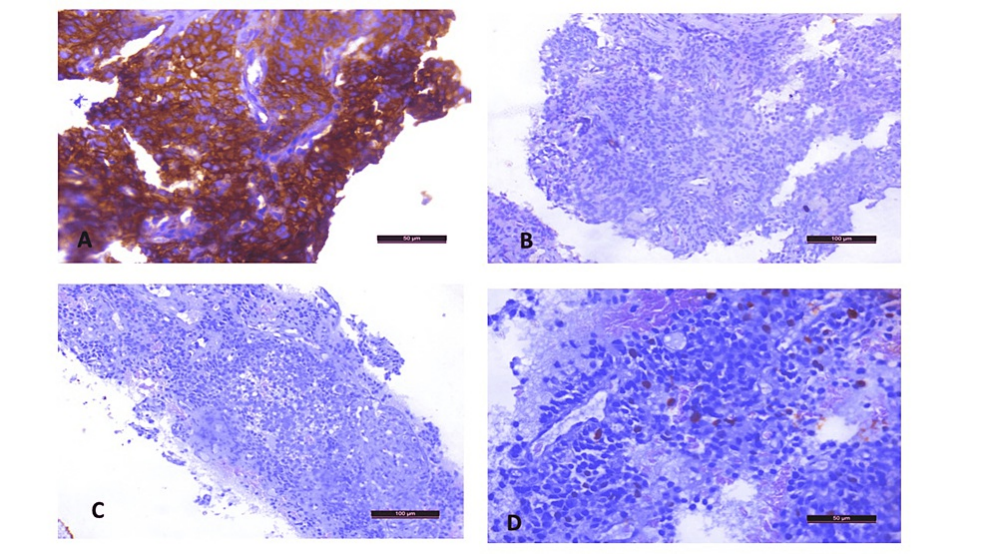
Immunohistochemistry images (A) Tumour cells show strong membranous positivity to CD99 (CD99; ×400) (scale 50 µm); (B) tumour cells do not show nuclear TTF1 positivity (TTF-1; ×200) (scale 100 µm); (C) no cytoplasmic chromogranin positivity (chromogranin; ×200) (scale 100 µm); (D) Ki67 proliferative index 35% (Ki67; ×400) (scale 50 µm)

Immunohistochemistry (IHC) revealed that the tumour cells showed positivity for CD99 (strong membranous staining). The tumour cells were negative for chromogranin, synaptophysin, TTF1, and AE1/AE3, thus excluding the possibility of neuroendocrine tumour and carcinoma of the lung, respectively (Figure 3A-3D). The Ki67 proliferative index was 35%. There was a possibility of Ewing's sarcoma, which was confirmed by the positivity of the IHC markers NKX2.2 and MIC2.

A final diagnosis of Ewing’s sarcoma/PNET of the thoracopulmonary region, i.e., Askin’s tumour, was given. The patient underwent five cycles of neoadjuvant chemotherapy using vincristine, doxorubicin, and cyclophosphamide and underwent surgical resection after achieving tumour burden reduction. The surgical outcome was satisfactory and the patient is on follow-up.

## Discussion

Haemoptysis can originate from disease of the airways, pulmonary parenchyma, or vasculature [5]. Disease of the airway can be inflammatory (acute or chronic bronchitis, bronchiectasis, cystic fibrosis) or neoplastic (bronchogenic carcinoma or bronchial carcinoid tumours). Parenchymal disease-causing haemoptysis may be localised (pneumonia, lung abscess, tuberculosis) or diffuse (Goodpasture syndrome, idiopathic pulmonary haemosiderosis). Vascular diseases associated with haemoptysis are pulmonary thromboembolic disease and arteriovenous malformations.

In 1979, Askin et al. published a compilation of 20 cases of childhood malignant small round cell tumours of the thoracopulmonary region, which later on was termed "Askin’s tumour" [3]. In their study, the age at diagnosis ranged from four months to 20 years, with a mean age of 14.5 years. A mass with or without pain was the most common presenting complaint, and dyspnoea and fever were other complaints, with none of the cases presenting with haemoptysis. Radiological findings showed masses ranging from 2-14 cm occupying the thoracopulmonary region, with some showing lung compression and rib erosion. Five cases showed rib erosion or destruction by the mass. On histopathological examination, the tumour cells were arranged in nests, compact sheets with fibrovascular stroma with accompanying necrosis. Classic Homer Wright rosettes were not identified, and tumour cells surrounding avascular matrix-forming pseudorosettes were seen in half of the cases. The mitotic activity ranged from 0-2 to 5-8 per 10 HPF. Studies show that the presence of glycogen granules (PAS positivity) is not a good indicator in differentiating Ewing’s sarcoma from neuroectodermal tumours [6]. In the indexed case, a 22-year-old presented with haemoptysis, with a tumour dimension of 13 cm and showing erosion of the 2nd rib. The size of the largest Askin’s tumour reported till date is 27 cm [7]. Pseudorosettes, mitosis (Ki-67%=35%), and PAS positivity for glycogen were seen.

In another review study on 11 cases of Askin’s tumour, the reported common presenting complaints were fever, cough, dyspnoea, chest wall tightness, and progressively enlarging chest wall tumour [8]. None of these 11 patients presented with haemoptysis. The immunohistochemical analyses showed that 10 out of the 11 cases showed positivity for CD99 and neuron-specific enolase (NSE). The indexed case also showed membranous intense CD99 positivity.

The differential diagnosis of small round cell tumours occurring in the thoracopulmonary region is Ewing’s sarcoma/PNET/Askin’s tumour, neuroblastoma, small cell osteosarcoma, synovial sarcoma, neuroendocrine tumours, and lymphoma (Table 1) [1,9].

**Table 1:** Differential diagnosis of malignant small round cell tumour in thoracopulmonary region [1,9]

Variables	Ewing’s sarcoma/PNET/Askin’s tumour	Neuroblastoma	Rhabdomyosarcoma	Osteosarcoma	Neuroendocrine tumours
Age	4 months - 20 years	Less than 10 years	Children and young adults	14-90 years	20-30 (carcinoid) 60-70 years (small cell carcinoma)
Clinical features	Chest wall mass with or without pain, fever, cough	Pain with palpable chest wall mass, syndromic presentation	Chest wall mass, dyspnoea, cough, pain	Palpable mass, dyspnoea, loss of weight	Chest pain, haemoptysis, paraneoplastic manifestations
Size of the tumour	2-27 cm	4-5 cm	3-16 cm	9-10 cm	Less than 3 cm
Histomorphology	Sheets, nests of uniformly arranged small, round cells forming pseudorosettes	Small, round blue cells forming Homer Wright rosettes	Fascicles and sheets of atypical spindle cells with brisk mitosis	Malignant osteoblasts with lacy osteoid deposition	Monomorphous population of small cells with finely granular chromatin and scant cytoplasm
IHC marker	CD99, MIC 2, NKX2.2, NSE (in many PNETs)	NSE, CD56, chromogranin, synaptophysin	Desmin, myogenin, MyoD1	IHC is of limited value BMP, osteocalcin, osteopontin, SATB2	Synaptophysin, chromogranin

Askin’s tumour seldom presents with haemoptysis and, to the best of our knowledge, this is the second case to be reported with haemoptysis [4]. In the previously reported case, the site of tumour was the bronchus intermedius. Haemoptysis in this case could have been due to the large size of the tumour ulcerating into the tracheobronchial tree with the rich vasculature of the tumour. The mild derangement of coagulation studies (reduced prothrombin index) and the possible relation between this group of tumours and their role in coagulopathies are yet to be explored.

Since the patient presented with haemoptysis, the initial suspicion was directed towards an intrapulmonary tumour. Studies reveal haemoptysis due to carcinoma of the lung to be still a common cause in patients aged less than 35 years [10]. Hence, AE1/AE3 and TTF1 were done to workup the possibility of carcinoma, which was negative in this case.

The CECT chest and PET CT scans helped in pointing out the origin of the tumour from chest wall. With subsequent correlation with histopathology and immunohistochemistry, helped the pathologist in arriving at the final diagnosis of Askin tumour, i.e., Ewing Sarcoma/PNET of the thoracopulmonary region.

In a retrospective study of 114 patients with non-metastatic Ewing’s sarcoma of the chest wall who were recipients of various treatment modalities including polychemotherapy, local radiotherapy, surgery, or a combination of radiotherapy and surgery, the overall survival was found to be about 60%. The recipients of pre-operative irradiation, though small in number, had no evidence of any local or systemic relapse [11]. The indexed patient underwent neoadjuvant chemotherapy and was surgically managed, with the surgical outcome being satisfactory.

## Conclusions

Haemoptysis is a rare clinical presentation of Askin’s tumour which can misdirect the pulmonologist to think in terms of a lung tumour arising from an intrapulmonary location rather than the chest wall/thoracopulmonary region. A young patient presenting with haemoptysis with a mass in the chest wall and rib erosion should prompt the suspicion of Ewing sarcoma/PNET of the thoracopulmonary region. A combined diagnostic approach utilising clinicoradiological findings in the context of histomorphological and judicious immunohistochemical analysis can help in arriving at an accurate diagnosis.
